# Combining DNA Damage Induction with BCL-2 Inhibition to Enhance Merkel Cell Carcinoma Cytotoxicity

**DOI:** 10.3390/biology9020035

**Published:** 2020-02-19

**Authors:** Wei Liu, Nathan A. Krump, Meenhard Herlyn, Jianxin You

**Affiliations:** 1 Department of Microbiology, Perelman School of Medicine, University of Pennsylvania, Philadelphia, PA 19104, USA; weiliu2@pennmedicine.upenn.edu (W.L.); nkrump@pennmedicine.upenn.edu (N.A.K.); 2 The Wistar Institute, 3601 Spruce Street, Philadelphia, PA 19104, USA; herlynm@wistar.org

**Keywords:** Merkel cell carcinoma, DNA damage induction, BCL2 inhibitor ABT-199

## Abstract

Merkel cell carcinoma (MCC) is a highly lethal skin cancer. MCC tumors rapidly develop resistance to the chemotherapies tested to date. While PD-1/PD-L1 immune checkpoint blockade has demonstrated success in MCC treatment, a significant portion of MCC patients are nonresponsive. Therefore, the pressing need for effective MCC chemotherapies remains. We screened a library of natural products and discovered that one compound, glaucarubin, potently reduced the viability of Merkel cell polyomavirus (MCPyV)-positive MCCs, while remaining nontoxic to primary human fibroblasts and MCPyV-negative MCC cell lines tested. Protein array and Western blot analyses revealed that glaucarubin induces DNA damage and PARP-1 cleavage that correlates with the loss of viability in MCC cells. However, high basal expression of the antiapoptotic factor BCL-2 allowed a subpopulation of cells to survive glaucarubin treatment. Previous studies have shown that, while targeting BCL-2 family proteins significantly decreases MCC cell viability, BCL-2 antisense therapy alone was insufficient to inhibit tumor growth in patients with advanced MCC. We discovered that treatment with an FDA-approved BCL-2 inhibitor in the context of glaucarubin-induced DNA damage led to near complete killing in multiple MCPyV-positive MCC cell lines that express high levels of BCL-2. The combination of DNA damage-induced apoptosis and BCL-2 inhibition thus represents a novel therapeutic strategy for MCPyV-positive MCCs.

## 1. Introduction

Merkel cell carcinoma (MCC) is a neuroendocrine carcinoma associated with Merkel cell polyomavirus (MCPyV) [1,2]. MCC metastasizes rapidly and is one of the most aggressive skin cancers [1,2], with a disease-associated mortality of 46% [3].

Despite increasing prevalence and poor patient outcomes, there are no effective chemotherapeutic treatments available for metastatic MCC [4,5]. Several prospective chemotherapies for MCC have had significant initial responses but rapidly lose efficacy due to chemoresistance [6]. Uncovering the mechanisms of resistance common to MCCs could lower rates of MCC recurrence after initial treatment and provide options for patients with advanced disease. Clinical trials exploring the use of PD-1/PD-L1 immune checkpoint inhibitors in combating metastatic MCC have yielded promising results, but a substantial portion of MCC patients are unresponsive to these immunotherapies [7,8,9,10,11,12,13]. Therefore, alternative therapies are needed for patients with advanced MCC.

MCPyV has a circular, double-stranded DNA genome of ~5 kb [1,2]. The viral genome is divided into early and late regions [1,14,15]. The early region encodes large T (LT), small T (sT), the 57kT antigen, and ALTO [1,16,17]. The late region encodes the capsid proteins, VP1 and VP2 [18,19,20].

In more than 80% of MCCs, MCPyV genomic DNA is clonally integrated into the tumor cell genome [2,21]. MCPyV integration typically results in tumor-specific truncation mutations that preserve the expression of sT and the N-terminal half of LT (LTT) [2,16]. Tumor-associated LTT retains the wild-type RB LxCxE binding motif, which sequesters and inactivates this tumor suppressor, and loses its C-terminal helicase domain that can induce DNA damage responses including p53 activation [16,22,23].

Compared to MCPyV-negative tumors, MCPyV-positive MCCs harbor very few somatic mutations [24,25], suggesting that the viral oncogenes play critical roles in driving tumor development. MCPyV-positive MCC cells are addicted to sT/LTT oncogenes and require their continued expression from the integrated viral genome to survive [26,27,28,29,30,31,32]. These findings suggest that MCPyV-positive and MCPyV-negative MCCs are supported by distinct oncogenic mechanisms and may require different treatment strategies. Also, compared to MCPyV-negative MCCs and other cancers that have high mutational burdens, MCPyV-positive MCCs likely have a narrower range of specific therapeutic targets. Therefore, identifying the correct combination of oncogenic targets could be a viable strategy to combat these virus-driven cancers.

In this study, we screened the compounds in the National Cancer Institute (NCI) Nature Products Set IV library and discovered that a natural product, glaucarubin, could specifically reduce viability of the MCPyV-positive MCC cell line MKL-1, but spare most of the MCPyV-negative MCC cells and healthy human skin cells. We found that glaucarubin treatment kills MKL-1 cells via accumulation of dsDNA breaks, but that highly expressed antiapoptotic BCL-2 allows a small population of cells to survive. The combination of glaucarubin-induced DNA damage and treatment with the FDA-approved BCL-2 inhibitor ABT-199 completely inhibits the proliferation of this subpopulation of cancer cells. In several other MCPyV-positive cell lines tested with high BCL-2 expression, including ABT-199, treatment greatly enhanced the cytotoxicity of glaucarubin alone. The synergy between DNA damage and BCL-2 inhibition in MCPyV-positive cell lines provides an explanation for why downregulation of BCL-2 mRNA alone was insufficient to impede tumor growth in patients with metastatic or regionally recurrent MCC [33]. The results discussed herein support a viable therapeutic strategy for MCPyV-positive MCCs in combining DNA damaging agents with BCL-2 inhibition.

## 2. Results

### 2.1. Identification of a Natural Product—Glaucarubin—That Can Specifically Inhibit the Growth of MCPyV-Positive MCC MKL-1 Cells

To identify candidate molecules with the potential to kill MCC cells, we performed cell cytotoxicity screening of the chemical compounds in the Natural Product Set IV provided by the NCI Developmental Therapeutics Program. This library contains 419 compounds isolated from natural products frequently consumed by humans. A subset of candidate compounds was identified in a luciferase reporter assay-based screen designed to isolate small molecules that can downregulate MCPyV oncogene transcription (data not shown). We then tested the cytotoxicity of those candidates in MKL-1 [34] cells and primary human dermal fibroblasts (HDFs), which served as archetypes for MCPyV-positive MCC and normally dividing cells, respectively.

**Figure 1:**
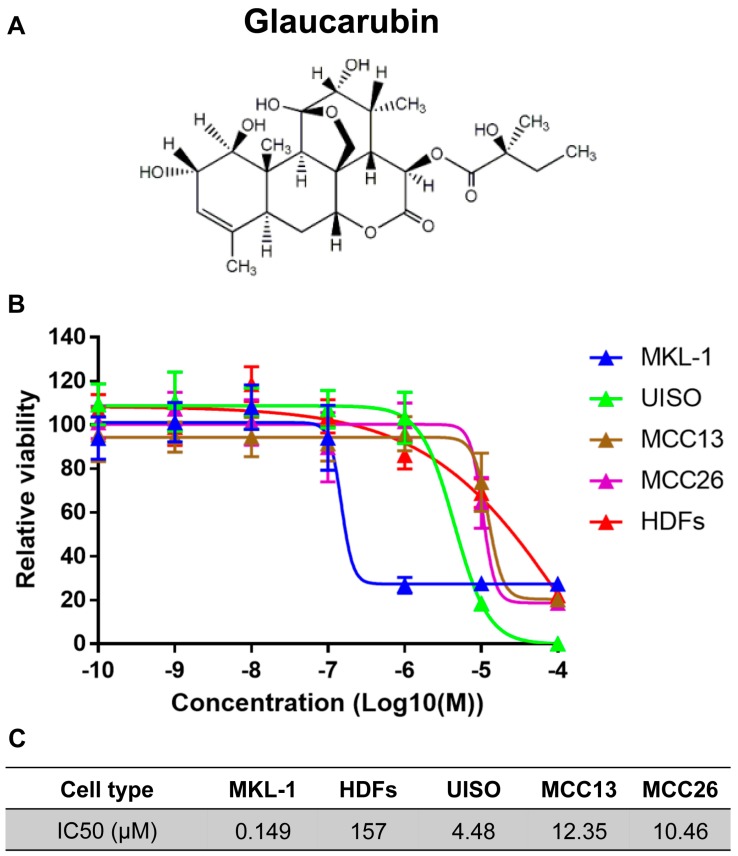
Natural product Glaucarubin specifically inhibits the growth of MCPyV-positive MCC MKL-1 cells. (**A**) Chemical structure of glaucarubin, a compound from the NCI Natural Product Set IV plate of identified to be toxic in MKL-1 MCC cells. (**B**) Assay of cell viability in the MCPyV-positive MCC cell line MKL-1; variant MCPyV-negative MCC cell lines UISO, MCC13, and MCC26; and primary HDFs subjected to the indicated concentrations for three days at 37 °C in 5% CO_2_. Cell viabilities were measured by cellTiter-GLO 3D cell viability assay (Promega). The growth curves were generated using GraphPad Prism software. (**C**) IC50 (μM) concentrations of glaucarubin for MCCs and HDF cells.

From this study, we discovered that one of the natural product-derived compounds, glaucarubin, could effectively kill MKL-1 with an IC50 of approximately 149 nM (Figure 1 and Figure S1). Glaucarubin has a much lower efficacy at killing the MCPyV-negative MCC cell lines UISO [35], MCC13 [36], and MCC26 [36], the IC50 values of which are about 30-, 83-, and 70-fold higher, respectively, compared to MKL-1 cells (Figure 1B,C). It is important to note that these MCPyV-negative cell lines have expression profiles that poorly represent MCC tumors in patients [37]. Since we were unable to acquire classical MCPyV-negative MCC lines for the present study, we cannot conclude whether glaucarubin may be toxic to those cells. When HDFs were treated with glaucarubin, we detected more than 1000-fold higher IC50 compared to the value obtained from MKL-1 cells (Figure 1B,C), suggesting that glaucarubin can kill MCPyV-positive MCC cells in concentration ranges that are nontoxic to healthy HDFs. In addition, the MCPyV-negative MCC cells and human primary keratinocytes (HFKs) were nearly as resistant to glaucarubin as HDFs (Figure 1B,C and Figure S2). The fact that both nonviral variant MCCs and HDFs required much higher concentrations of glaucarubin to achieve the same cytotoxicity as MKL-1 suggested that glaucarubin activates a cell death pathway that is only present in MCPyV-positive MCC cells. Notably, ~20% of MKL-1 cells remained viable, even at the highest glaucarubin concentration tested (Figure 1B and Figure S1), implying that this subpopulation is resistant.

### 2.2. Analysis of Glaucarubin Derivatives for the Potency in Killing MCPyV-Positive MKL-1 Cells

**Figure 2:**
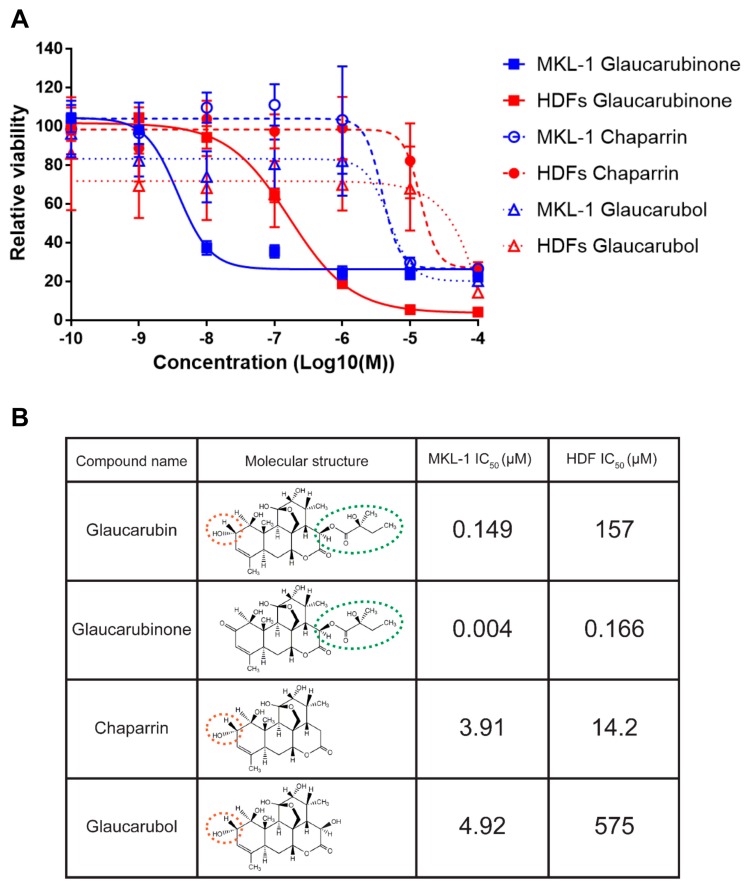
Glaucarubin derivatives inhibit the growth of MCC and HDF cells. (**A**) Cell viability assay of MCPyV-positive MCC cell line MKL-1 (blue) and HDFs (red) treated with the indicated concentrations of glaucarubin analogs 72 h post-treatment. (**B**) Table highlighting the IC50s of each compound from (**A**) in MKL-1 and HDF cells. Molecular structures of each compound are included for the purpose of relating features of the compounds to phenotypes in cells. For example, the ester-linked moiety featured on the lactone of glaucarubin (circled with a green dotted line) appears necessary for the cytotoxicity in MKL-1 cells, while the hydroxyl group (circled with an orange dotted line) results in less cytotoxicity in HDFs than a carbonyl group at the same position.

To explore the mechanism of action for glaucarubin cytotoxicity on MKL-1 cells, we sought to determine whether glaucarubin-related compounds were also toxic (Figure 2). We compared the capacity of these compounds to inhibit the growth of MKL-1 cells and normal HDFs. We also collated the proliferation data with the respective chemical structures of these compounds in order to identify the chemical moieties essential for glaucarubin cytotoxicity. Two of the compounds tested, chaparrin and glaucarubol, had 978- and 1230-fold higher IC50 values on MKL-1, respectively (Figure 2). Since these compounds lack the ester-linked moiety featured on the lactone of glaucarubin, we reasoned that this side chain is important for the deleterious function of glaucarubin observed in MKL-1 (Figure 2B). In support of this hypothesis, glaucarubinone, which contains the same ester-linked domain as glaucarubin, showed the strongest potency in killing MKL-1 with an IC50 of nearly 4 nM, which is 40-fold lower than the IC50 of Glaucarubin on MKL-1 (Figure 2). However, compared to glaucarubin, glaucarubinone also has a much lower IC50 (approximately 166 nM) for HDFs, suggesting that it is too toxic to be a good candidate for drug development. Since glaucarubin was the only compound that greatly hampered MKL-1 viability, but not HDFs, we proceeded with this original candidate for functional analysis.

### 2.3. Protein Array Characterization of Cellular Genes Targeted by Glaucarubin

**Figure 3:**
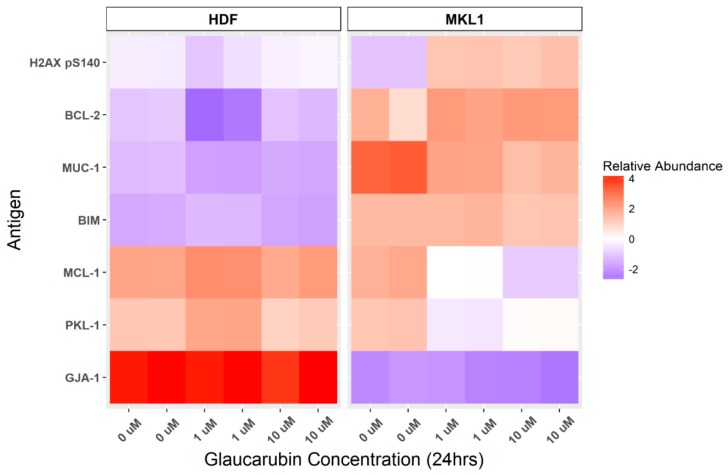
Protein array identification of glaucarubin-affected genes. HDFs and MKL-1 cells were treated with DMSO or glaucarubin at the indicated concentrations. Duplicate cell lysates were harvested at 24 h post-treatment for each condition and subjected to RPPA analysis. The linear signal of each slide was normalized to actin for that same sample. Antigens that had a linear expression level greater than 2 for at least one of the conditions in MKL-1 cells were analyzed. Antigens increased or decreased in abundance after glaucarubin treatment were ranked in order of their greatest abundance change relative to that in HDFs. The six antigens with the greatest increase or decrease are pictured in the heat map. Heat map relative abundance scores are a visualization of actin-normalized, Log 2, median-centered signal for each sample. MUC-1, Mucin 1; BIM, Bcl-2-like protein 11, PKL-1, polo-like kinase 1; GJA-1, Gap junction alpha-1 protein.

To explore possible explanations for the discrepancy between MKL-1 and HDF sensitivity to glaucarubin, and to identify targetable cellular factors underlying its mechanism of action, we applied reverse-phase protein microarray (RPPA) to probe glaucarubin-induced changes in the abundance of antigens with documented functions in cancer development and progression (Figure 3, Table S1). We treated MKL-1 and HDFs with 1 µM or 10 µM of glaucarubin dissolved in DMSO or DMSO alone in duplicate. The lysates were harvested from these samples at 24 h post-treatment; prior to the onset of significant cell death in MKL-1 at the concentrations tested. RPPA plates were probed with stringently validated antibodies to over 300 cancer-related protein targets (Table S1). Antigens abundant in MKL-1 that experienced the greatest shift upon glaucarubin-treatment relative to that in HDFs included key factors in DNA damage responses and apoptosis (Figure 3). γH2A.X, a marker of DNA double-strand breaks and repair, is significantly elevated in MKL-1, but not in HDFs, after treatment with glaucarubin (Figure 3). Two anti-apoptotic proteins, BCL-2 and MCL-1, are also differentially expressed in MKL-1 in the presence of glaucarubin, but are maintained at a low level in HDFs in each condition tested (Figure 3). While both genes have relatively high basal expression in MKL-1, increasing the glaucarubin concentration further increases BCL-2 level while reducing the MCL-1 level (Figure 3).

Previous studies have shown that prosurvival BCL-2 and MCL-1 are highly expressed in up to 85% and 88% of MCCs, respectively [38,39,40]. Between these two genes, BCL-2 plays a particularly important role in oncogenesis by inhibiting apoptosis [41]. High BCL-2 expression has been shown to support better the growth and survival of MCC [42,43]. In the context of these previous studies, our findings suggest that glaucarubin may trigger DNA damage-induced apoptosis in MKL-1, yet further stimulation of BCL-2 levels by glaucarubin may contribute to the resistance observed in ~20% of the cells surviving the treatment (Figure 1B).

### 2.4. Glaucarubin Induces a Cell Death Pathway in MCPyV-Positive MCC

**Figure 4:**
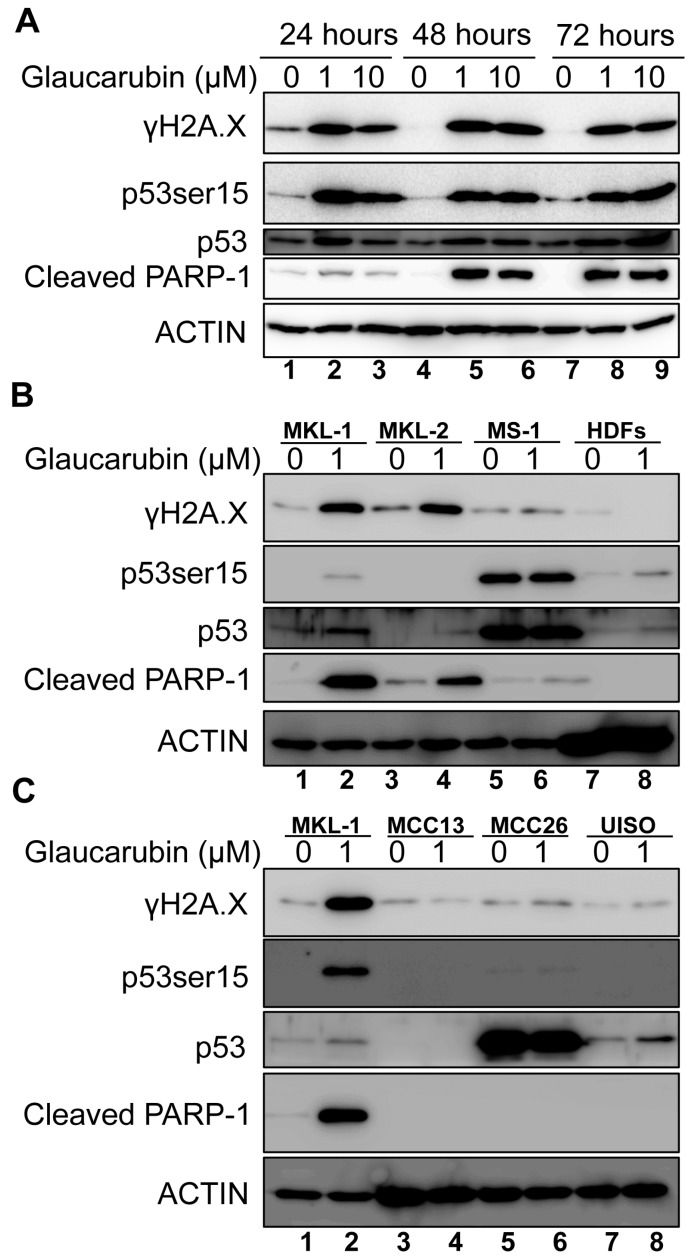
Glaucarubin-associated toxicity correlates with γH2A.X accumulation and PARP-1 cleavage. (**A**) MKL-1 cells were treated with DMSO or glaucarubin dissolved in DMSO for final concentrations equaling 1 µM or 10 µM, respectively. At 24, 48, and 72 h, total cell lysates were harvested and immunoblotted with the indicated antibodies. ACTIN was used as a loading control. (**B**) Lysates from MCPyV-positive cell lines MKL-1, MKL-2, and MS-1, as well as HDFs were treated with 1 µM of glaucarubin dissolved in DMSO or DMSO alone for 48 h and harvested for immunoblot analysis. (**C**) Same as (**B**) except with MCPyV-negative cell lines MCC-13, MCC-26, and UISO.

To test the hypothesis that glaucarubin kills MCPyV-positive MCC cells by stimulating DNA damage-induced cell death response, we first validated our RPPA results in MKL-1 cells treated with glaucarubin via Western blot (Figure 4A). Indeed, the γH2A.X level was elevated in MKL-1 24 h after treatment with either 1 or 10 µM glaucarubin and remained elevated 72 h post-treatment, indicating that DNA damage is induced in these cells (Figure 4A). By immunofluorescent staining, we discovered that glaucarubin induces robust double-strand DNA breaks, very few single-strand DNA breaks, and no reactive oxygen species (ROS) in MKL-1 cells (Figure S3). Glaucarubin treatment does not increase the γH2A.X level in HDFs, which also corroborated our protein array data (Figure 4B). The lack of γH2A.X accumulation in glaucarubin-treated HDFs may be because these cells are able to quickly repair any DNA insults induced by the compound.

Next, we determined whether glaucarubin treatment induces DNA damage in other MCPyV-positive cell types (Figure 4B and Figure S4). Glaucarubin treatment also resulted in the accumulation of γH2A.X in MCPyV-positive MCC cell lines MKL-2 [44], PeTa [45] and BroLi [31], but failed to do so in MS-1 [31] and WaGa [31], over the course of the 48-h treatment (Figure 4B and Figure S4). MS-1 cells do not accumulate γH2A.X after glaucarubin treatment, likely because they overexpress inactive p53 [44], which, in the presence of low-level, persistent DNA damage, could prevent cell cycle arrest and subsequent γH2A.X accumulation via caspase-mediated chromosomal degradation. Variant MCPyV-negative MCC cell lines MCC13, MCC26, and UISO also do not accumulate γH2A.X in the presence of glaucarubin (Figure 4C). That the presence or absence of γH2A.X accumulation with glaucarubin treatment correlated with susceptibility or resistance to the drug toxicity with respect to different MCC cell lines (Figure 1C) suggested to us that glaucarubin induces cell death in MCPyV-positive MCC cells by inducing DNA damage and downstream signaling pathways.

To test our hypothesis that canonical events downstream of DNA damage and γH2A.X accumulation incurred by glaucarubin were at the root of its cytotoxicity, we analyzed the p53 activation status and poly (ADP-ribose) polymerase (PARP-1) cleavage in each cell type in the presence of glaucarubin. p53 serine 15 phosphorylation, a marker for activated p53, is dramatically induced in glaucarubin-treated MKL-1, PeTa, and BroLi, where robust γH2A.X accumulation is observed (Figure 4A,B and Figure S4). Significant PARP-1 cleavage, representing late-stage apoptotic progression, was also induced in these MCPyV-positive MCC cells at 48 h post-treatment. The MCC cell lines that were resistant to glaucarubin toxicity did not exhibit PARP-1 cleavage, nor phosphorylation of p53 at serine 15 with the exception of MS-1, which overexpresses inactive p53 [46] (Figure 4B,C). WaGa cells, which express wild-type p53, did not develop γH2A.X marks nor PARP-1 cleavage in the presence of glaucarubin (Figure S4). Based on the levels of induction of γH2A.X and PARP-1 cleavage, we infer from these data that MKL-1, MKL-2, PeTa, and BroLi are sensitive to glaucarubin-induced apoptotic cell death. Interestingly, MKL-2 cells exhibited PARP-1 cleavage despite lacking detectable p53 expression and are therefore incapable of apoptosis through the canonical DNA damage pathway. This suggests that glaucarubin may induce apoptosis by another mechanism in these cells. The heterogeneity even among MCPyV-positive MCC cell lines underscores the need to target cell-survival factors that are common among many MCCs. Compared to MKL-1, glaucarubin-treated MKL-2 had a lower degree of PARP-1 cleavage, which could potentially be attributed to an undetectable level of activated p53 present in these cells. These results suggest that the mechanism of action of glaucarubin in MCPyV-positive MCC cells involves the stimulation of DNA damage and apoptosis.

### 2.5. BCL-2 Function Supports the Resistance of MCPyV-Positive MCC Cells to Glaucarubin Killing

**Figure 5:**
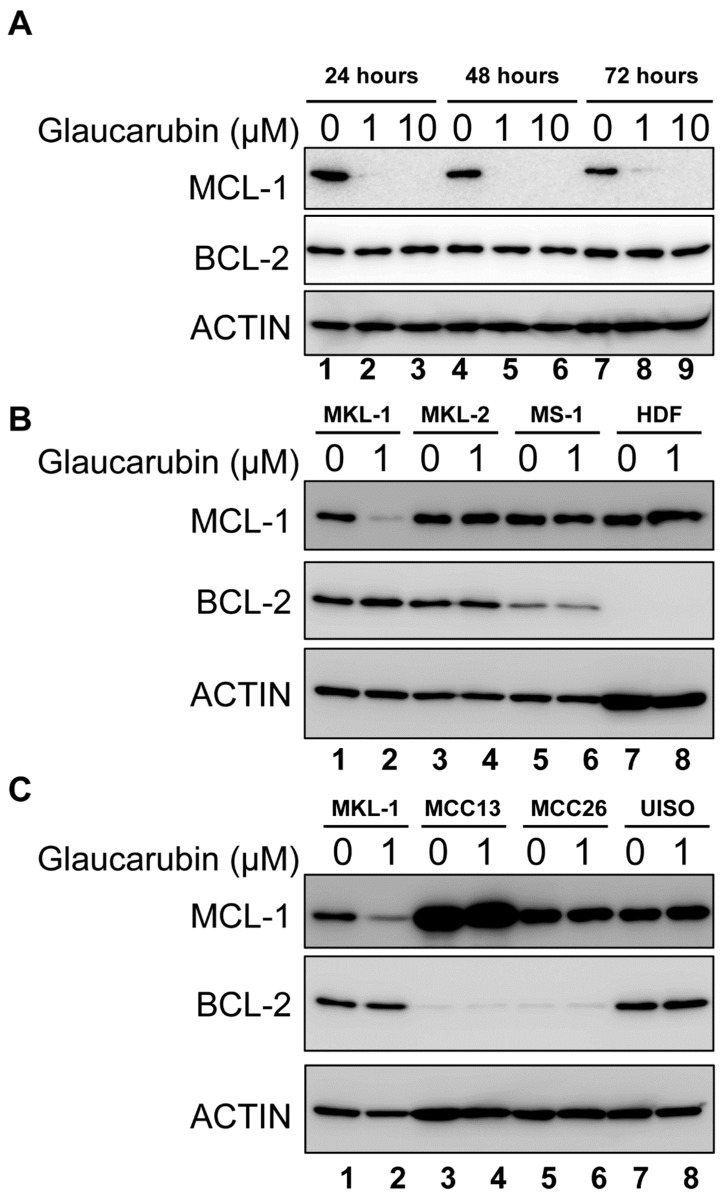
Glaucarubin does not affect BCL-2 expression. (**A**) MKL-1 cells were treated with DMSO or glaucarubin dissolved in DMSO for final concentrations of 1 µM or 10 µM, respectively. Total cell lysates were harvested after 24, 48, and 72 h of treatment and analyzed by Western blotting using the indicated antibodies. (**B**) Western blot of total cell lysates from MCPyV-positive MCC cell lines MKL-1, MKL-2, MS-1, and primary HDFs treated with DMSO or glaucarubin dissolved in DMSO for a final concentration of 1 µM for 48 h. (**C**) Similar data as shown in (**B**) except with total cell lysates from MCPyV-negative MCC cells MCC13, MCC26, and UISO.

In MKL-1 treated with glaucarubin, we consistently observed that nearly 20% of the cells remained alive, even after treatment with very high concentration of glaucarubin (Figure 1B). From the protein array analysis, we discovered that antiapoptotic proteins BCL-2 and MCL-1 are highly expressed in MKL-1 compared to HDFs (Figure 3). After glaucarubin treatment, the MCL-1 level was dramatically reduced in MKL-1, but the BCL-2 level was further elevated (Figure 3). These observations suggest that robust BCL-2 expression may allow some of the MKL-1 cells to escape glaucarubin killing (Figure 1B). To examine this possibility and validate the protein array observations, we performed a Western blotting analysis of BCL-2 and MCL-1 in MKL-1 treated with increasing doses of glaucarubin. Confirming the findings from the protein array, MCL-1 diminished after glaucarubin treatment, while the BCL-2 level remained elevated (Figure 5A). However, in the other MCPyV-positive MCC cell lines tested, such as MKL-2, MS-1, PeTa, WaGa, and BroLi, both the MCL-1 and BCL-2 expression remained unaltered by glaucarubin treatment (Figure 5B and Figure S4). That glaucarubin only reduces the MCL-1 level in MKL-1 cells implies that other MCPyV-positive MCCs may be more resistant to glaucarubin as a result of MCL-1 anti-apoptotic function. The consistently high BCL-2 expression across each of the MCPyV-positive MCC lines that incur DNA damage in the presence of glaucarubin suggests that it is a dominant anti-apoptotic factor. Conversely, HDFs and variant MCPyV-negative MCC cell lines, MCC13 and MCC26, do not express a detectable level of BCL-2. The only MCPyV-negative MCC cell line that shows robust BCL2 expression is UISO, which also maintains unchanged BCL-2 levels upon glaucarubin treatment. This result suggested that high BCL-2 expression in MCPyV-positive cells likely supports survival after glaucarubin treatment.

### 2.6. Combined Treatment of an FDA-Approved BCL-2 Inhibitor and Glaucarubin Leads to Complete Killing of MCPyV-Positive MCC Cells

**Figure 6:**
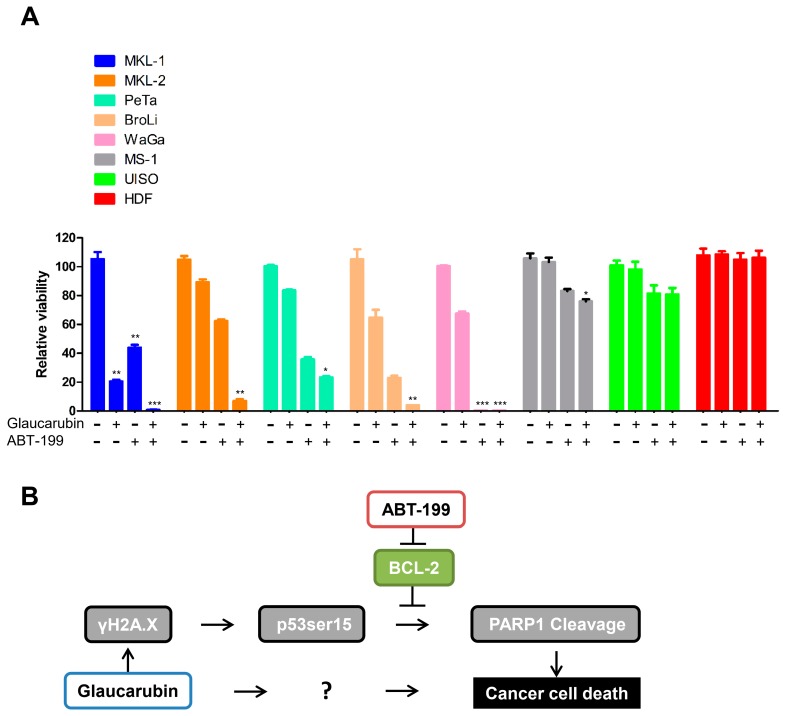
Targeting BCL2 function using an FDA approved inhibitor to achieve better killing of MCC cells. (**A**) Cell viability assay of MCC cells and primary HDFs treated with DMSO or the indicated concentrations of glaucarubin, ABT-199, or both for 72 h at 37 °C in 5% CO_2_. An asterisk (*) signifies the number of standard deviations from the mean. *P* < 0.05, ** *P* < 0.01, *** *P* < 0.001. (**B**) Proposed working schematic of effects induced by glaucarubin in MCPyV-positive MCC cell lines. MCCs can develop resistance to this cell death pathway by failing to repress BCL-2. Inhibition of BCL-2 by ABT-199 can circumvent this resistance mechanism. The question mark denotes an unknown mechanism underlying the sensitivity of MCPyV-positive MCC cells to glaucarubin.

Given that glaucarubin readily induces DNA damage in MCPyV-positive MCCs such as MKL-1, MKL-2, PeTa, and BroLi, but failed to inhibit BCL-2 in any of the cell types tested, we reasoned that BCL-2 activity in these MCPyV-positive MCC cells could confer resistance to complete loss of viability after glaucarubin treatment. Thus, we tested whether a combination of glaucarubin and the FDA-approved BCL-2 inhibitor, ABT-199, could circumvent resistance in those MCC cells (Figure 6A). As expected, neither glaucarubin nor ABT-199 alone was sufficient to kill all MKL-1, MKL-2, PeTa, and BroLi cells. In combination, however, glaucarubin and ABT-199 efficacy was greatly increased in all of these cell lines (Figure 6A). In WaGa cells, ABT-199 is responsible for the dominant effect that kills nearly 100% of the cells. MS-1, which lacks a DNA damage phenotype upon glaucarubin treatment (Figure 4 and Figure 5), remained unaffected when only treated with glaucarubin. BCL-2 inhibition, alone or in combination with glaucarubin, also had a minor impact on MS-1 viability, likely because these cells do not express a high level of BCL-2 (Figure 5 and Figure 6). The viability of normal primary HDFs was not impacted by any of the treatments (Figure 6A), which is consistent with our earlier observations that no γH2A.X accumulation is observed in these cells after glaucarubin treatment and that there is no detectable level of BCL-2 expressed in these cells (Figure 5B and Figure 6).

## 3. Discussion

Currently, there are no effective chemotherapeutic strategies for combating metastatic MCCs, and those that have been attempted have failed to produce durable responses. The recently developed PD-1/PD-L1 immune checkpoint inhibitors have demonstrated promising results but, in many cases, the responses are temporary [8,10,11,21,47]. Therefore, alternative therapeutics are needed for treating advanced-stage MCCs.

In this study, we performed a compound screening and identified the natural product glaucarubin as a potent inhibitor that can specifically repress the growth of MCPyV-positive MCC cells. Glaucarubin is a crystalline glycoside extracted from the tropical plant *Simarouba glauca* [48]. We discovered that glaucarubin could specifically inhibit the growth of MCPyV-positive cells such as MKL-1 at low concentrations (with an IC50 of nearly 149 nM), without introducing much toxicity for control MCPyV-negative MCC and healthy skin cells, even at very high concentrations (IC50 ranges from 4.48 to 157 µM).

To search for possible molecular mechanisms underlying glaucarubin cytotoxicity observed in MCPyV-positive MCC cells, we performed a protein array analysis of putative oncogenes, tumor suppressors, and metastatic factors in normal healthy HDFs and MKL-1 cells after glaucarubin treatment. We found that γH2A.X is one of the most significantly increased antigens in MKL-1 cells after glaucarubin treatment, but it remained unchanged in HDFs under the same conditions (Figure 3 and Figure 4). We also found that γH2A.X induction and PARP-1 cleavage in MCPyV-positive MCC cells correlates with the induction of a well-characterized anticancer, cell death effector pathway (Figure 4 and Figure S4).

An analysis of the MCPyV-positive and -negative MCC cell lines demonstrated that the antiproliferative activity of glaucarubin largely hinges on its ability to induce DNA-damage-associated cell death, though other pathways may be involved (Figure 4 and Figure S4). For example, MCPyV-positive MKL-1 cells, which accumulate γH2A.X and subsequent PARP-1 cleavage after glaucarubin treatment, are highly responsive to glaucarubin killing. Glaucarubin treatment induces a similar set of apoptotic markers, but to a lesser degree in other MCPyV-positive MCC cell lines, MKL-2, PeTa, and BroLi, and predictably does not kill these cells with the same efficacy (Figure 6A). It is possible that MKL-1 cells are especially susceptible to glaucarubin treatment because the antiapoptotic factor MCL-1 is uniquely downregulated by glaucarubin in these cells (Figure 3 and Figure 5). Normal HDFs, MCPyV-positive MCC MS-1 cells, and MCPyV-negative MCC13, MCC26, and UISO cells, all of which do not show accumulation of γH2A.X upon glaucarubin treatment, are consistently resistant to glaucarubin (Figure 1C). In these cells, glaucarubin either does not induce DNA damage, or induces a level of DNA damage that can be repaired or tolerated. WaGa cells present an exception to our observations in that glaucarubin fails to induce γH2A.X or PARP-1 cleavage but they still appear partially sensitive to glaucarubin cytotoxicity (Figure 6A). This may be a result of some other mechanism. For example, WaGa grow in a single-cell suspension rather than aggregates like other MCPyV-positive MCC lines; therefore, they may take up more of the drug or be susceptible to downregulation of MUC-1, a membrane glycoprotein that was observed in the MKL-1 RPPA (Figure 3).

Although glaucarubin is effective at killing MCPyV-positive cells, nearly 20% of the MKL-1 cells remain viable even after treatment with as much as 0.1 mM of glaucarubin. RPPA analysis showed that the antiapoptotic regulator, BCL-2, is highly expressed in MKL-1 cells (Figure 3). BCL-2 inhibits apoptosis, in part, by blocking p53-induced cell death [49,50]. Canonically, activated p53 can circumvent antiapoptotic activity by upregulating several proapoptotic genes such as BAX, Noxa, BID, and PUMA [51,52,53,54], all of which can directly or indirectly downregulate BCL-2 function [55,56,57]. In addition, p53 can directly repress transcription of BCL-2 [57,58,59] (Figure 6B). Both RPPA and Western blotting analysis revealed that BCL-2 maintains robust expression after MCPyV-positive MCC cell lines were treated with glaucarubin (Figure 3 and Figure 5, and Figure S4), indicating that even in the cell lines where p53 is activated, it is not sufficient to reduce the BCL-2 level (Figure 6B). This finding also suggests that persistent BCL-2 expression in MCPyV-positive cells might enable escape from the mitochondrial apoptosis pathway (Figure 6B). Constitutive BCL-2 expression could then contribute to the residual viability of glaucarubin-treated MCPyV-positive MCC cells that we observed in Figure 1B and Figure 6A. Previous efforts and our observations suggest that a combination of DNA damage and BCL-2 inhibition could effectively kill BCL-2-high, MCPyV-positive MCCs with low mutational burdens (Figure 6B). Indeed, dual treatment with glaucarubin and BCL-2 inhibitor ABT-199 resulted in the killing of almost all of the MCPyV-positive MCC cells tested (Figure 6A). Furthermore, although WaGa cells did not incur marks of DNA damage, they are so dependent on BCL-2 expression for survival that ABT-199 treatment alone is sufficient for complete killing (Figure 6A) In contrast, combined treatment was well tolerated in cell types in which these two molecular signatures are not detected, as in MS-1, UISO, and HDFs (Figure 6A). Others have shown that, while inhibition of BCL-2 family proteins can significantly induce MCC cell death [43], BCL-2 antisense therapy alone was insufficient to inhibit tumor growth in patients with advanced MCC [33]). Therefore, another important aspect of our finding is revealing that DNA damage-induced apoptosis provides an opportunity to enhance the efficacy of BCL-2 inhibitor for treating MCPyV-positive MCCs.

Our results suggest that p53 may contribute to the cell death induced by glaucarubin in some MCPyV-positive MCC cell lines such as MKL-1, PeTa, and BroLi (Figure 4 and Figure S4). However, in some other virus-associated MCC cells, such as WaGa, no p53 activation was observed (Figure S4). In addition, MCPyV-positive MKL-2 cells are responsive to glaucarubin treatment without expressing a detectable level of p53 (Figure 4). These findings again suggest that, rather than the p53 activity in the tumor cells, DNA damage induced by glaucarubin is the key determining factor that controls its cytotoxicity. The additional pathways underlying the sensitivity of these MCC cells to glaucarubin-mediated DNA damage remains to be investigated in future studies (Figure 6B). Nevertheless, our findings suggest that combining a DNA-damage-inducing agent, such as glaucarubin, with BCL-2 inhibitors represents a novel strategy for treating MCPyV-positive MCCs with high BCL-2 expression.

Glaucarubin has been used as an herbal medicine for treating intestinal amoebiasis for more than six decades [48,60,61]. In addition, ABT-199 is an FDA-approved anticancer drug. The clinically documented safety of glaucarubin and ABT-199 in humans highlights their potential for rapid translation towards clinical application of the dual treatment for curing other cancers with high BCL-2 levels and low mutational burdens, and therefore intact intrinsic apoptotic pathways.

The current standard of care for initial presentation of MCC is resection of tissue at the primary site, and irradiation of the resected site as well as draining lymph nodes. MCCs are considered highly radiation-sensitive tumors, as evidenced by the observation that disease is eliminated in many patients after radiation treatment alone. Recurrent and especially metastatic MCC, however, have much worse prognoses and are more difficult to treat. Given evidence of high expression of BCL-2 in many MCCs, and its potential as an escape mechanism from DNA-damage-mediated cell death, it would seem that BCL-2 inhibition may be best applied in combination with existing radiation treatments at first diagnosis. MCPyV-positivity and basal BCL-2 expression can be determined from primary tumor biopsies with relative ease and are likely predictors of the success of this combination. The great challenge of treating late-stage cancers with diverse mutational backgrounds lends credence to an aggressive treatment strategy for the primary tumor. ABT-199 treatment combined with radiation of the primary tumor and lymph nodes could prove effective at eliminating MCPyV-positive MCCs that would otherwise produce chemotherapeutically resistant metastases. Chemotherapeutically sensitizing tumors to DNA-damage-induced death may also make it possible for radiation specialists to reduce the dosages and concomitant toxicity needed for effective treatment.

## 4. Materials and Methods

### 4.1. Cell Culture

MCPyV-positive MCC cell lines MKL-1, MKL-2 [44], MS-1 [31], PeTa [45], WaGa [31], and BroLi [31]; MCPyV-negative MCC cell lines UISO [35], MCC13 [36], and MCC26 [36]; and HDFs were used in the drug screening. The MCC cell lines were grown in RPMI 1640 supplemented with 20% fetal calf serum, penicillin, and streptomycin at 37 °C in humidified air containing 5% CO_2_. The remaining cell lines were grown in Dulbecco’s modified Eagle’s medium supplemented with 10% fetal calf serum. HDFs and human primary keratinocytes were isolated and cultured as described [62].

### 4.2. Compounds

All compounds were obtained from NCI Developmental Therapeutics Program Open Chemical Repository (http://dtp.cancer.gov). All glaucarubin-derived compounds were obtained from NCI/DTP Open Chemical Repository. BCL2 inhibitor ABT-199 (Cat. NO. 16233) was purchased from Cayman Chemical (Ann Arbor, MI, USA).

### 4.3. Cytotoxicity Screening

Cells were seeded at a density of 5000 cells in 100 µL of medium per well. Cells were incubated at 37 °C in humidified air containing 5% CO_2_ for 72 h with drugs. Cell viability was measured with CellTiter-Glo 3D (Promega, Madison, WI, USA) following the manufacturer’s instructions. The validity of CellTiter-Glo results in measuring cell viability was confirmed by WST-1 assays (Roche, Basel, Switzerland) in pilot studies.

### 4.4. Western Blot Analysis

Western blotting was performed as described previously [62]. The primary antibodies used in this study include anti-γH2A.X (1:1000, 2577S, Cell Signal Technology, Danvers, MA, USA), anti-p53 (1:500, sc-6243, Santa Cruz Biotechnology, Dallas, TX, USA), anti-p53ser15 (1:1000, 9286S, Cell Signal Technology, Danvers, MA, USA), anti-cleaved PARP1 (1:1000, 5625S, Cell Signal Technology, Danvers, MA, USA), anti-MCL1 (1:1000, 39224S, Cell Signal Technology, Danvers, MA, USA), anti-BCL2 (1:1000, 2872S, Cell Signal Technology, Danvers, MA, USA), anti-ACTIN (1:150,000, MAB1501, Millipore, Burlington, MA, USA). The secondary antibodies used were HRP-linked anti-rabbit IgG (1:4000, 7074S, Cell Signaling Technology, Danvers, MA, USA) and HRP-linked anti-mouse IgG (1:4000, 7076S, Cell Signaling Technology, Danvers, MA, USA).

### 4.5. Reverse Phase Protein Lysate Array (RPPA)

HDFs and MKL-1 treated in duplicate with DMSO or glaucarubin dissolved in DMSO for final concentrations equaling 1 µM or 10 µM, respectively. After 24 h, cell lysates were harvested for each condition. A total of 50 µg protein per sample was subjected to RPPA analysis using a set of validated antibodies targeting 304 antigens with documented roles in cancer. The RPPA assay was performed by the staff members of the MD Anderson Cancer Center core facility. All antibodies used in the RPPA assay were previously validated by Western blotting [63].

### 4.6. Statistical Analyses

Statistical analysis was performed using the unpaired *t*-test of GraphPad Prism software (Version 5.0) (San Diego, CA, USA) to compare the data from the control and experimental groups. A two-tailed *p* value of <0.01 was considered statistically significant.

## 5. Conclusions

A natural product, glaucarubin, induces dsDNA breaks, specifically in p53 wild-type MCPyV-positive MCCs. It is capable of significantly reducing the viability of those cells at the nanomolar range in vitro. High BCL-2 expression, however, enables a portion of p53 wild-type/virus-positive MCC cells to escape apoptotic cell death. Killing across multiple cell lines with these attributes is nearly total when glaucarubin-induced DNA damage is combined with treatment with an FDA-approved BCL-2 inhibitor, ABT-199.

## Supplementary Materials

The following are available online at https://www.mdpi.com/2079-7737/9/2/35/s1, Figure S1: WST-1 viability assay to detect glaucarubin cytotoxicity in MKL-1 cells. Figure S2: HFK cell viability assay. Figure S3. Glaucarubin induces mostly double-strand DNA break in MCPyV-positive MCC cells. Figure S4. The mechanism for glaucarubin-induced cell death in MCPyV-positive MCC cells. Table S1: Raw RPPA data of glaucarubin-treated MKL-1 cells and primary HDFs.
