# Supplementary material for: Combining DNA Damage Induction with BCL-2 Inhibition to Enhance Merkel Cell Carcinoma Cytotoxicity
Source: Biology (Basel). 2020 Feb 19;9(2):35. doi: 10.3390/biology9020035 (PMC7168258; doi:10.3390/biology9020035)
Supplement: Supplementary file 1 [file biology-09-00035-s001.zip › Figures.docx]

**Figure S1**





**Figure S1**: **WST-1 viability assay to detect glaucarubin cytotoxicity in MKL-1 cells.** MKL-1 cells were seeded at a density of 5000 cells in 100 µl of medium per well of a 96-well plate. Cells were incubated at 37°C in humidified air containing 5% CO2 for 72 hours with DMSO or 1µM glaucarubin. Cell viability was measured using WST-1 assays (Roche) following the manufacturer’s instructions.

**Figure S2**





**Figure S2: HFK cell viability assay.** HFKs were seeded at a density of 5000 cells in 100 µl of medium per well of a 96-well plate. The cells were incubated with DMSO or the indicated concentrations of glaucarubin at 37°C in humidified air containing 5% CO2 for 72 hours. Cell viability was measured using CellTiter-Glo 3D (Promega) following the manufacturer’s instructions.

**Figure S3**


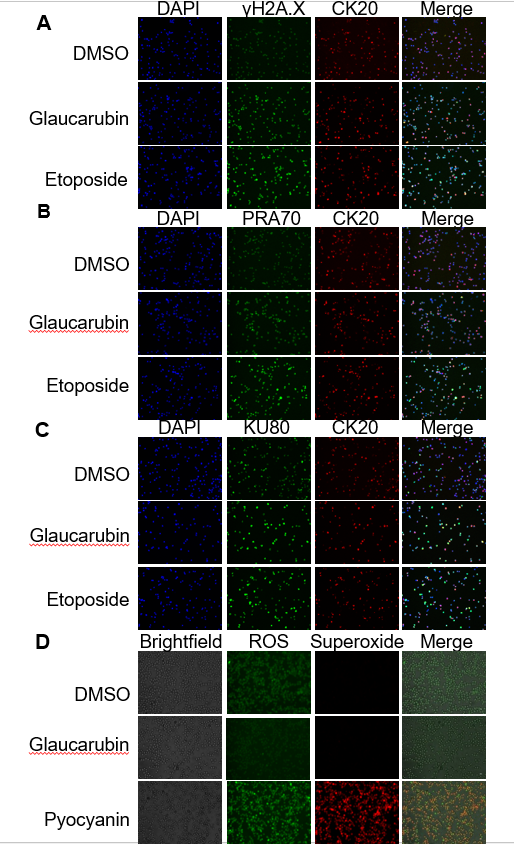


**Figure S3. Glaucarubin induces mostly double-strand DNA break in MCPyV-positive MCC cells. (A-C)** MKL-1 cells were treated with DMSO, glaucarubin, or etoposide dissolved in DMSO at 1 µM final concentrations. After one hour, cells were immunofluorescent stained with the indicated antibodies. **(D)** MKL-1 cells were treated with DMSO or glaucarubin dissolved in DMSO at 1 µM final concentrations. Pyocyanin treatment was used as a positive control. After one hour, ROS and superoxide levels were measured using ROS/Superoxide Detection Assay Kit (Abcam).

**Figure S4**


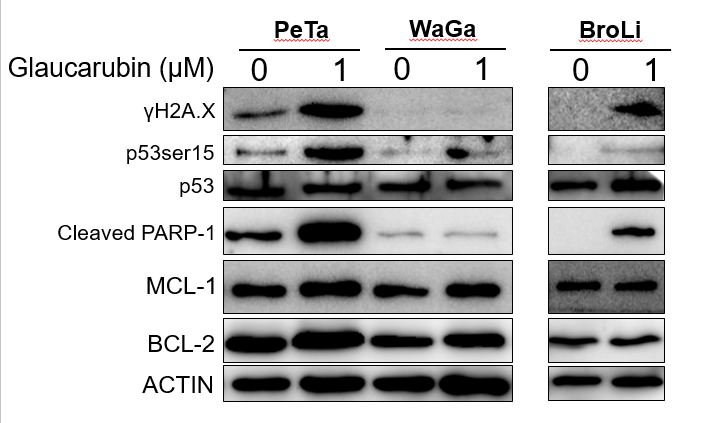


**Figure S4. The mechanism for glaucarubin-induced cell death in MCPyV-positive MCC cells.** MCPyV-positive MCC cell lines PeTa, WaGa and BroLi were treated with DMSO or glaucarubin dissolved in DMSO at a final concentration of 1 µM. After 48 hours, total cell lysates were harvested and immunoblotted with the indicated antibodies. ACTIN was used as a loading control.
